# GC4S: A bioinformatics-oriented Java software library of reusable graphical user interface components

**DOI:** 10.1371/journal.pone.0204474

**Published:** 2018-09-20

**Authors:** Hugo López-Fernández, Miguel Reboiro-Jato, Daniel Glez-Peña, Rosalía Laza, Reyes Pavón, Florentino Fdez-Riverola

**Affiliations:** 1 ESEI—Escuela Superior de Ingeniería Informática, Universidad de Vigo, Ourense, Spain; 2 CINBIO—Centro de Investigaciones Biomédicas, Universidad de Vigo, Vigo, Spain; 3 SING Research Group, Galicia Sur Health Research Institute (IIS Galicia Sur), SERGAS-UVIGO, Spain; 4 Instituto de Investigação e Inovação em Saúde (I3S), Universidade do Porto, Porto, Portugal; 5 Instituto de Biologia Molecular e Celular (IBMC), Porto, Portugal; Swiss Institute of Bioinformatics, SWITZERLAND

## Abstract

Modern bioinformatics and computational biology are fields of study driven by the availability of effective software required for conducting appropriate research tasks. Apart from providing reliable and fast implementations of different data analysis algorithms, these software applications should also be clear and easy to use through proper user interfaces, providing appropriate data management and visualization capabilities. In this regard, the user experience obtained by interacting with these applications via their Graphical User Interfaces (GUI) is a key factor for their final success and real utility for researchers. Despite the existence of different packages and applications focused on advanced data visualization, there is a lack of specific libraries providing pertinent GUI components able to help scientific bioinformatics software developers. To that end, this paper introduces GC4S, a bioinformatics-oriented collection of high-level, extensible, and reusable Java GUI elements specifically designed to speed up bioinformatics software development. Within GC4S, developers of new applications can focus on the specific GUI requirements of their projects, relying on GC4S for generalities and abstractions. GC4S is free software distributed under the terms of GNU Lesser General Public License and both source code and documentation are publicly available at http://www.sing-group.org/gc4s

## Introduction

The importance of bioinformatics and computational biology in research today is leading to the development of a growing number of complex bioinformatics tools and software packages. In this context, these applications must solve two important needs: the provision of reliable implementations of data analysis algorithms, as well as convenient interfaces enabling their effective use for conducting appropriate research [1]. Indeed, several studies highlighting the importance of applying proper software engineering practices and methodologies to systematically develop high-quality bioinformatics software have been published [2,3]. Similarly, some authors have contributed with valuable and helpful recommendations for the appropriate development of effective scientific software [4,5]. The concept of user experience when developing such bioinformatics applications is becoming increasingly important, and some studies also provide useful guidelines for improving the development of user friendly bioinformatics software [1,6]. All the approaches discussed above highlight how the usability of different types of bioinformatics applications can be improved by adopting well-established practices in usability and software engineering.

Taking the previously discussed facts into account, several frameworks, platforms and libraries for different programming languages (e.g. Java, R, Python, C++, etc.) and complementary bioinformatics areas (e.g. proteomics, genomics, etc.) were recently released with the goal of facilitating the development of successful bioinformatics applications [7–11]. This plethora of alternatives provides a reliable basis for developing novel algorithms and data analysis functionalities, allowing developers to extend them and reuse existing code. Several libraries and applications focused on scientific and biological data visualization [7,12–14] are also available. A notable example is the JSparklines library [15], which provides the capability of visualizing numbers in Java Swing tables, making use of sparklines. These data visualization libraries certainly help developers create new applications and provide excellent features to end-users. However, other aspects ensuring an effective user experience (e.g. the creation of input dialogs, configuration panels and other user interaction steps) are inadequately supported due to a lack of specific resources providing reusable Graphical User Interface (GUI) components able to help developers in the same way as many of the aforementioned frameworks and libraries.

In the same line of previous approaches, we also contributed to this collection with AIBench [16], a Java application framework for scientific software development. Since its first release, it has been successfully used to create different bioinformatics software such as LA-iMageS [17], Mass-Up [18], S2P [19,20], BioAnnote [21], BEW [22], @Note [23], ADOPS [24], DPD [25] or MLibrary [26], among others, covering a wide range of application areas including, but not limited to, proteomics, genomics and text mining. By providing the most common functionalities present in typical scientific applications, such as user parameter collection, logging, multithreaded execution, experiment repeatability, workflow management, and automatic GUI generation, AIBench allows developers to focus on the specific application logic. The wide range of successfully released bioinformatics software based on AIBench demonstrates that our framework has provided an effective alternative to increase productivity while developing high-quality source code [27].

The development of AIBench applications always relies on the straightforward implementation of three different but complementary types of programmable components: *operations*, *data types* and *views*. However, our long-term experience in the field has proved that GUI (a key feature in any deployment) should be improved further. As previously stated, AIBench automatically constructs the user interface by generating (*i*) application menus based on declared operations and (*ii*) input dialogs for obtaining required operation parameters. However, the *view* components, which must be developed in Java Swing, are the responsibility of the programmer. In this regard, we noticed that these kinds of components were being copied and pasted between code bases, including non-AIBench based applications. In doing so, developers were missing an opportunity for reusing GUI elements by sharing them between code bases and saving time in future projects.

Bearing all that in mind, and with the specific goal of overcoming these shortcomings, we initially designed, and later developed and tested, GC4S (GUI Components for Swing), an open source software library that aims to help programmers developing Java Swing based scientific applications. GC4S is a collection of high-level, extensible, bioinformatics-oriented and reusable Java GUI elements created by using Swing and SwingX low-level components. By publishing this library, developers of new applications can exclusively focus on the specific GUI requirements of their projects, relying on GC4S for generalities. This way, they can also benefit from bug fixes, updates, and improvements in GC4S. To the best of our knowledge, there is currently no other open-source, free library offering such functionalities.

The remainder of the paper is structured as follows: while Section 2 introduces our novel GC4S library discussing implementation details and essential concepts, Section 3 illustrates the usefulness of GC4S for scientific software development by presenting three complementary case studies showing how GC4S can be easily integrated with the AIBench framework or independently used for speeding up the development process of coding standalone applications. Section 4 discusses the main contributions of this work as well as the lessons we learned in the last few years. Finally, Section 5 summarizes the main conclusions extracted from this contribution and outlines future research work.

## The GC4S library: Implementation and components

### Implementation

GC4S 1.2 is implemented in Java 8 as an Apache Maven project to automatically build the library. The source code of the project is freely available at https://github.com/sing-group/GC4S under a GNU LGPL 3.0 License (http://www.gnu.org/copyleft/lgpl.html). This project contains six main Maven modules: (*i*) *gc4s*, containing the general-purpose GC4S components; (*ii*) *gc4s-genomebrowser*, containing a component to visualize genomes and biological data; (*iii*) *gc4s-heatmap*, containing a component to create heat map representations of numerical data; (*iv*) *gc4s-jsparklines-factory*, containing classes to facilitate the creation of JSparkLines renderers [14]; (*v*) *gc4s-multiple-sequence-alignment-viewer*, containing a component to visualize multiple sequence alignments; and (*vi*) *gc4s-statistics-tests-table*, containing a component to display datasets in customizable tables and automatically compute p-values and q-values to compare the conditions in them. Moreover, for each of these six modules, there is a *-demo module containing demos and examples.

At the implementation level, components in the main modules (hereafter, GC4S components) can be grouped into three main categories: (*i*) *extensions*, that is, components that enhance existing components in order to add functionalities; (*ii*) *high-level components*, that is, components that provide new functionalities by grouping other existing components; and (*iii*) *utilities*, components that provide service methods (e.g. builders) or resources (e.g. icons).

### Components

From the programmer’s perspective, GC4S components serve three main purposes: (*i*) retrieve user input; (*ii*) display data or results; and (*iii*) provide different programming utilities. According to these functionalities, they are classified as Input components, Output (or Visualization) components or Utility components, respectively. Regarding the structure of the source code of the library, Table 1 shows the top-level GC4S packages, reflecting how components are grouped according to their function. In addition, Supporting Information S1 Table provides a comprehensive list of the entire GC4S library, providing a brief description of each component. Extensive Javadoc documentation is also available at http://www.sing-group.org/gc4s/javadoc. Supporting Information S1 Document provides basic documentation about the usage of the library in Maven-based projects as well as examples of some components of the *gc4s* module.

**Table 1:** GC4S library structure. Packages are prefixed by org.sing_group.gc4s, which is ommitted to avoid redundancy.

Package	Description
.dialog	Provides components related to the creation of different types of dialogs.
.dialog.wizard	Provides components related to the creation of Wizard dialogs or assistants.
.event	Provides extensions of interfaces and classes related to different types of events fired by AWT (Abstract Window Toolkit) components.
.input	Provides components related to the retrieval of different types of user inputs.
.input.combobox	Provides components related to the retrieval of user input using combo boxes.
.input.csv	Provides components related to the retrieval of CSV (comma-separated values) configurations.
.input.filechooser	Provides components related to file selection.
.input.list	Provides components related to the retrieval of user input using lists.
.input.text	Provides components related to the retrieval of user input using text fields.
.jsparklines	Provides components related to the creation of different types of JSparkLines renderers.
.msaviewer	Provides components related to the visualization of multiple alignment sequences.
.statistics	Provides components related to the visualization of statistical tests tables.
.ui	Provides components related to the creation of user interfaces and layouts.
.ui.icons	Provides icons and utilities related to icons.
.ui.menu	Provides components related to the creation of menus.
.ui.tabbedpane	Provides components related to the creation of tabbed panes.
.ui.text	Provides components related to the creation of text components.
.utilities	Provides classes offering different functionalities that cannot be grouped in other packages (dialog, event, input, ui or visualization).
.utilities.builder	Provides builder classes to facilitate the creation of components.
.visualization	Provides components related to data visualization.
.visualization.heatmap	Provides components related to heat map visualization.
.visualization.table	Provides components related to table visualization.
.visualization.table.csv	Provides components related to table visualization of CSV data.

To demonstrate the utility and purpose of the library under consideration, this section illustrates, by way of example, how different components work and can be easily used. As mentioned above, the main purpose of GC4S is to provide a reliable set of generic GUI components that can be efficiently reused in scientific applications development, allowing programmers to focus on concrete problem details.

### Genome browser

The *gc4s-genomebrowser* module provides a graphical component called GenomeBrowser to visualize genomes and different types of biological data, in a manner similar to the web Human Genome Browser at UCSC [28]. This component relies on the PileLine library [29] for parsing the genomic files.

**Fig 1:**
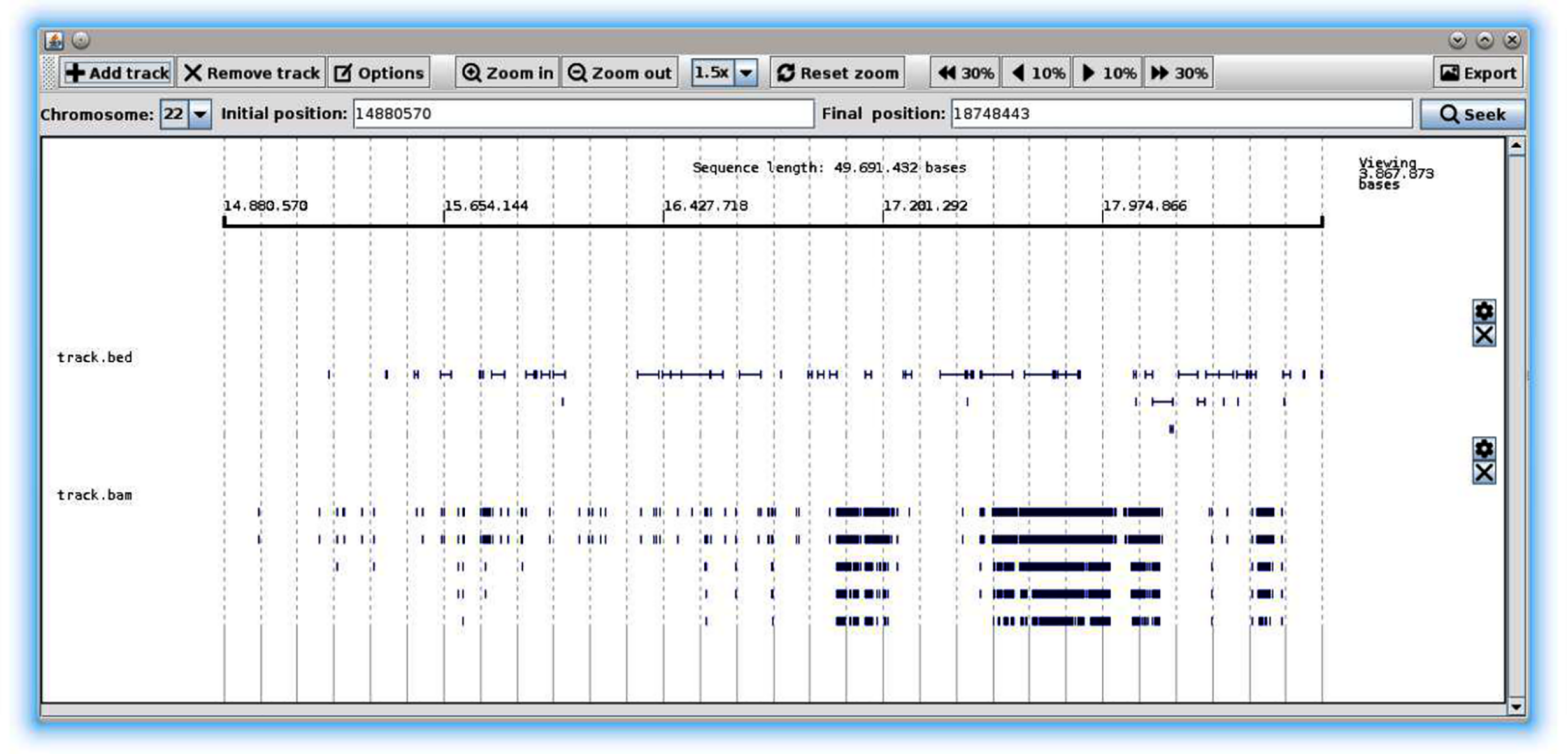
The GenomeBrowser component showing two tracks.

**Fig 2:**
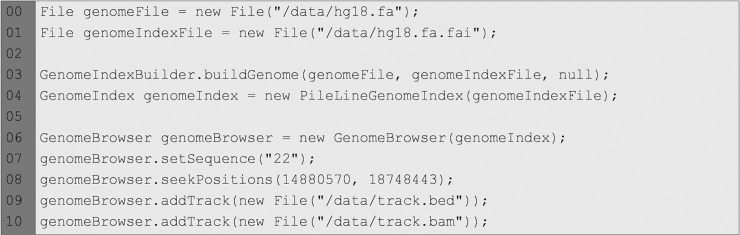
Code snippet showing the instantiation of a GenomeBrowser. Also, the range and chromosome of interest and two tracks are set programmatically.

The GenomeBrowser component in Fig 1 shows the visualization of a reference genome with two *tracks*. As the code snippet in Fig 2 shows, this component must be instantiated by providing an object of a class implementing the GenomeIndex PileLine interface. In this example, the genome index is created using the GenomeIndexBuilder provided by PileLine and then instantiated by creating a PileLineGenomeIndex object. Alternatively, the genome index may be created using samtools [30] and then instantiated by an object of the FaiGenomeIndex class. The *tracks* containing biological data are read from genomic position files including standard bam (http://samtools.github.io/hts-specs/SAMv1.pdf), pileup (http://samtools.sourceforge.net/pileup.shtml), BED (http://genome.ucsc.edu/FAQ/FAQformat#format1), GFF (http://genome.ucsc.edu/FAQ/FAQformat#format3) or VCF (http://www.internationalgenome.org/wiki/Analysis/vcf4.0/) formats. In the case of bam files, the corresponding .*bai* index must exist in the same directory as the provided bam file.

The toolbar in the upper area allows users to interactively browse their data by specifying the genome range and chromosome of interest, zooming in or out, and so on. Additionally, other settings such as colour, background colour, maximum number of depth levels, and other options, can be customized for each track.

### Heat map

The *gc4s-heatmap* module provides a graphical component called JHeatMap to visualize numerical data matrices as heat maps, graphical representations where the individual values are represented as colours.

**Fig 3:**
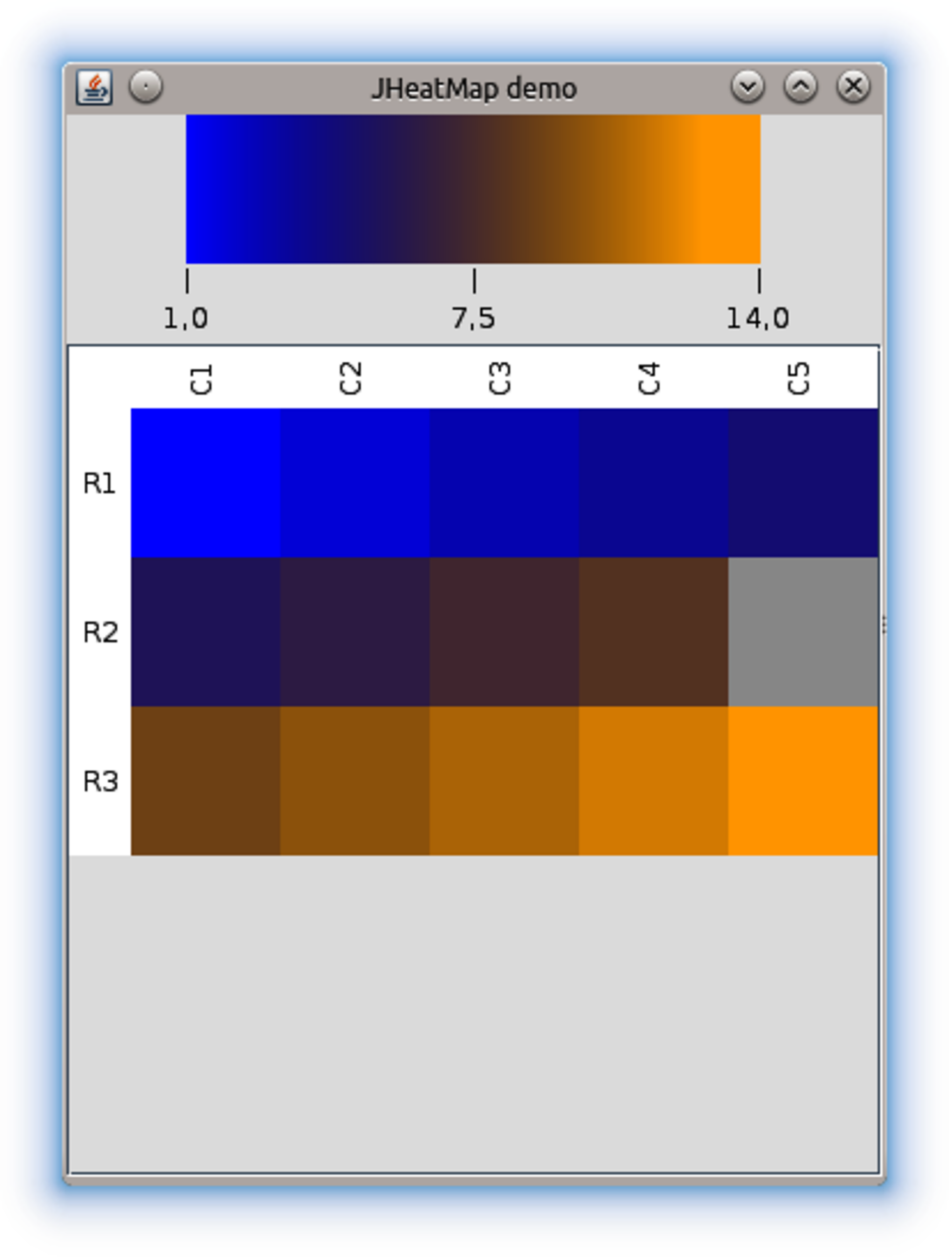
The JHeatMap component.

**Fig 4:**
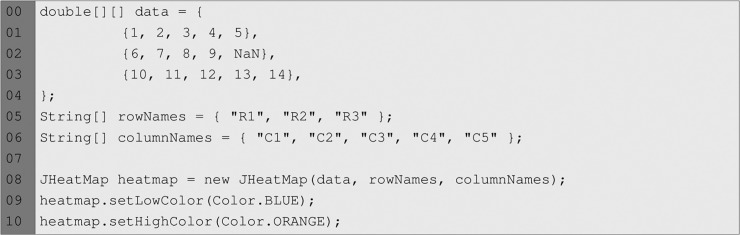
Code snippet showing the instantiation of a JHeatMap. Also, the colours that must be used to create the colour gradient are set programmatically.

The JHeatMap component in Fig 3 shows the visualization of a data matrix with three rows and five columns. As the code snippet in Fig 4 shows, this component must be instantiated by providing a data matrix and two arrays specifying rows and columns names. Alternatively, it can be instantiated by providing a JHeatMapModel object. Also, the colours that must be used to create the gradient are set programmatically in this example. This component has the mouse wheel zooming feature enabled by default, but it can be programmatically disabled.

Based on this core component, it is provided the JHeatMapPanel component that presents a JHeatMap along with a toolbar for controlling different visualization options. These options are: (*i*) changing the colours of the colour gradient, (*ii*) changing the minimum and maximum values that must be used to compute the colour gradient, (*iii*) changing the font settings, (*iv*) transforming the data matrix (e.g. apply row centring, log transform data, etc.), (*v*) establishing the visible rows and columns, and (*vi*) exporting the heat map as image. This component must be instantiated by providing a previously created JHeatMap component.

### Multiple Sequence Alignments Viewer

The *gc4s-multiple-sequence-alignment-viewer* module provides a graphical component called MultipleSequenceAlignmentViewerPanel to display multiple sequence alignment data.

**Fig 5:**
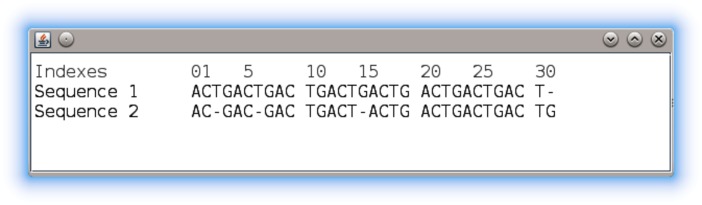
The MultipleSequenceAlignmentViewerPanel component.

**Fig 6:**
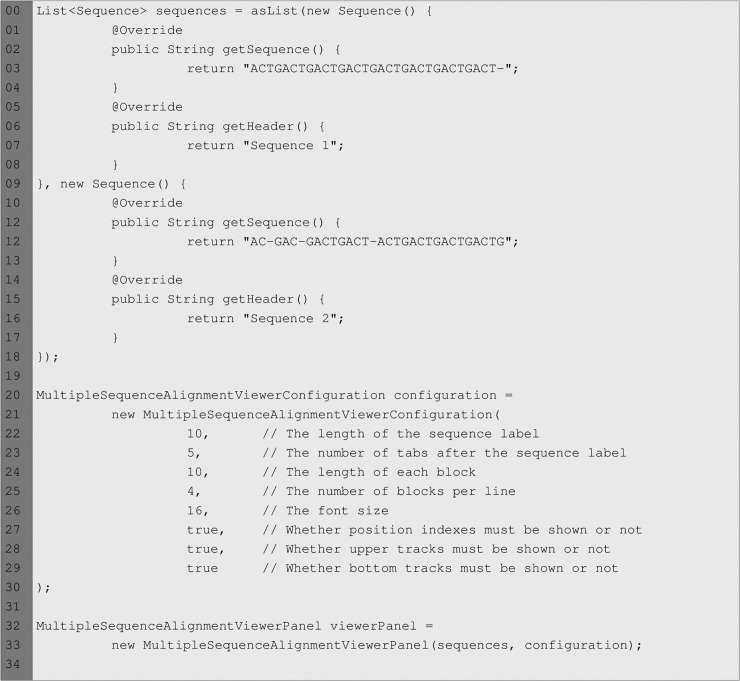
Code snippet showing the instantiation of a MultipleSequenceAlignmentViewerPanel. The initial visualization settings are also passed to the constructor.

The MultipleSequenceAlignmentViewerPanel component in Fig 5 shows the visualization of two sample sequences. As the code snippet in Fig 6 shows, this component must be instantiated by providing a list of objects that implement the Sequence interface. Also, a configuration object can be passed to set the initial visualization settings. If this object is not provided, the default settings are used.

**Fig 7:**
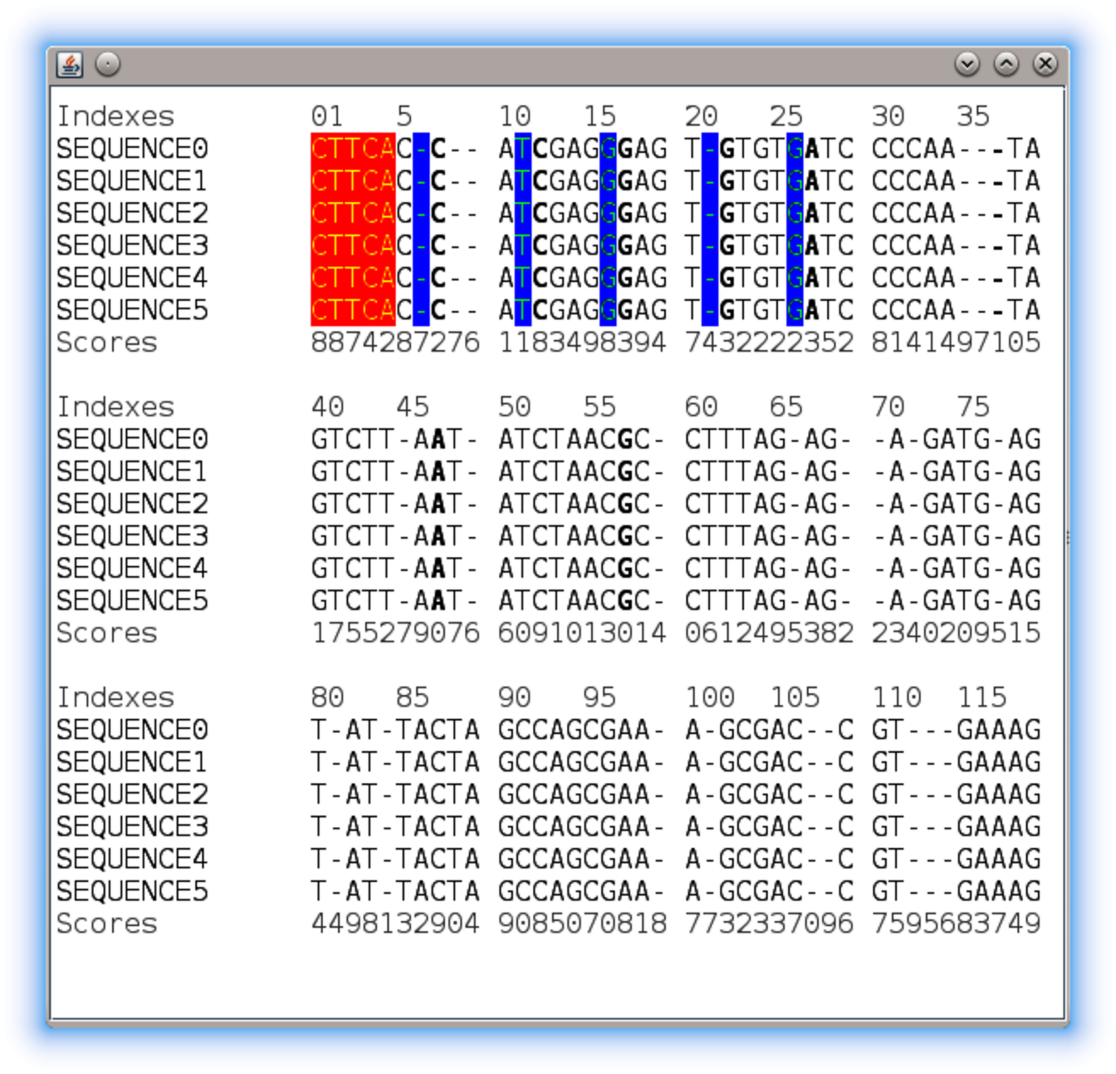
The MultipleSequenceAlignmentViewerPanel component configured with a renderer and a model that adds a track named *Scores*.

When this visualization is used, two features are usually required: (*i*) the possibility of highlighting specific sequence positions (e.g. to point out a specific amino acid or nucleotide as a result of a biological analysis, etc.), (*ii*) the possibility of adding additional information in form of tracks that are placed before or after the sequences (e.g. to add some kind of score associated with each sequence position). In GC4S, these can be achieved by the usage of SequenceAlignmentRenderer and MultipleSequenceAlignmentTracksModel objects, respectively. These objects can be added to the viewer constructor so that they can be used to show additional information. Fig 7 shows an example of this component configured with a renderer and a model that adds a track named *Scores*.

Based on this core component, it is provided the MultipleSequenceAlignmentViewerControl component that presents a MultipleSequenceAlignmentViewerPanel along with a toolbar with different options for the end-users of the visualization. These options are: (*i*) selecting the tracks model and renderer that are being shown, (*ii*) changing the viewer configuration through a GUI dialog, and (*iii*) exporting the view as image or HTML document. Additional advanced examples of these two components are provided in the corresponding *demo* module.

### Statistics Tests Table

The *gc4s-statistics-tests-table* module provides a graphical component called StatisticsTestTable to visualize tabular datasets and automatically compute p-values and q-values for each row in order to compare the conditions in them.

The table is generic, meaning that it can be used to display any type of data. The table retrieves the data from generic datasets implementing the Dataset interface, which also has a default implementation called DefaultDataset. The statistical tests used to compute p-values in each row to compare the conditions in the dataset must be compatible with the type of data under consideration. This means that a table created with a Dataset<Boolean> should receive a Test<Boolean>. The package org.sing_group.org.gc4s.statistics.data.tests of this module provides test implementations for Boolean (Chi-squared test, Chi-squared test with Yates' correction, Randomization test, Fisher’s Exact test), Number (several t-tests and ANOVA), and String (Chi-squared test) data. Developers using the library can develop their own tests by simply implementing the Test interface.

**Fig 8:**
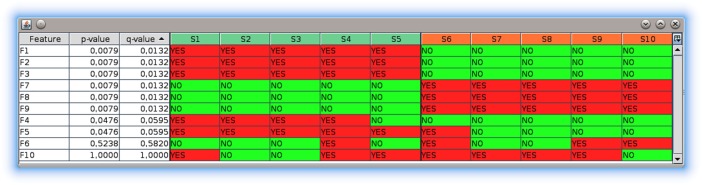
The StatisticsTestTable component.

**Fig 9:**
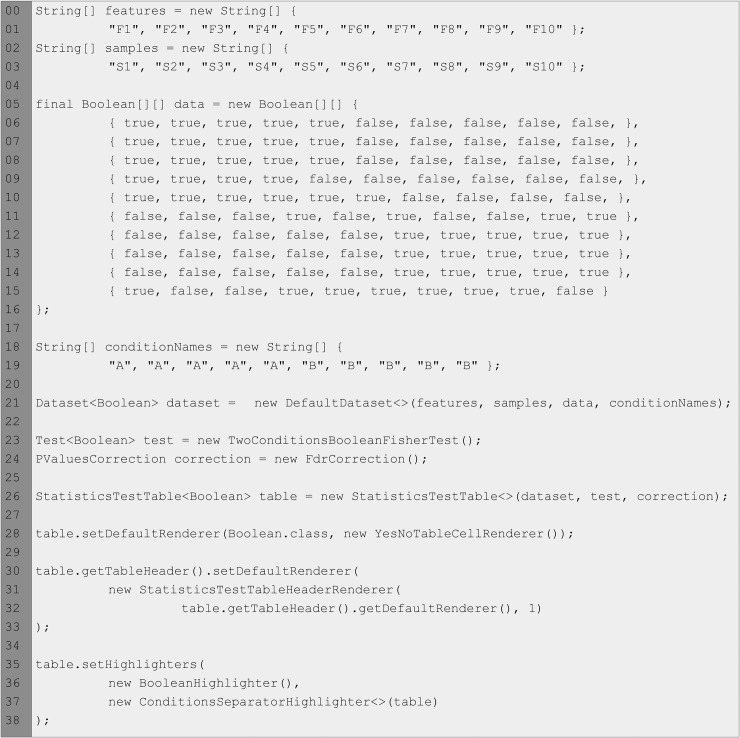
Code snippet showing the instantiation and configuration of a StatisticsTestTable.

The StatisticsTestTable component in Fig 8 shows the visualization of a tiny dataset of Boolean values with two conditions, ten samples and ten features (for illustrative purposes, the size is small because it is meant to be a minimal example; advanced examples with bigger datasets can be found at the corresponding *demo* module). As the code snippet in Fig 9 shows, this component must be instantiated by providing a Dataset and a Test objects for the same type. Optionally, an object that implements the Correction interface can be provided in order to have one additional column displaying the corrected p-values obtained from the test, as it is the case of this example. After its instantiation, the table is also configured to: (*i*) set a cell renderer that displays text “YES” or “NO” instead of true and false; (*ii*) set a header cell renderer that displays sample names using different colours depending on their associated conditions and draws a left border at the first column of each condition to enhance the visual distinction between conditions; (*iii*) set a highlighter that uses different colours for the cell values (true in red and false in green), and (*iv*) set a highlighter that draws a left border at the first sample of each condition to enhance the visual distinction between conditions. These classes are also included in this module, thus they are reusable by the programmers of the library in the same way that we use them in the demos.

The time needed for the computation of the p-values and q-values can vary depending on the dataset size and the statistical tests used, so a ProgressEventListener can be added to the table in order to receive progress events from the component. Based on this core component, it is provided a StatisticsTestTablePanel component that presents a StatisticsTestTable along with a progress bar to monitor the progress computation. This component is instantiated in the same way than the basic table and the table used internally can be obtained programmatically so that it can be fully configured and controlled.

## Case studies

Two AIBench-based applications and a regular standalone application were selected as case studies to illustrate how GC4S helped in the development of actual bioinformatics software.

### AIBench-based applications: S2P and DEWE

**Fig 10:**
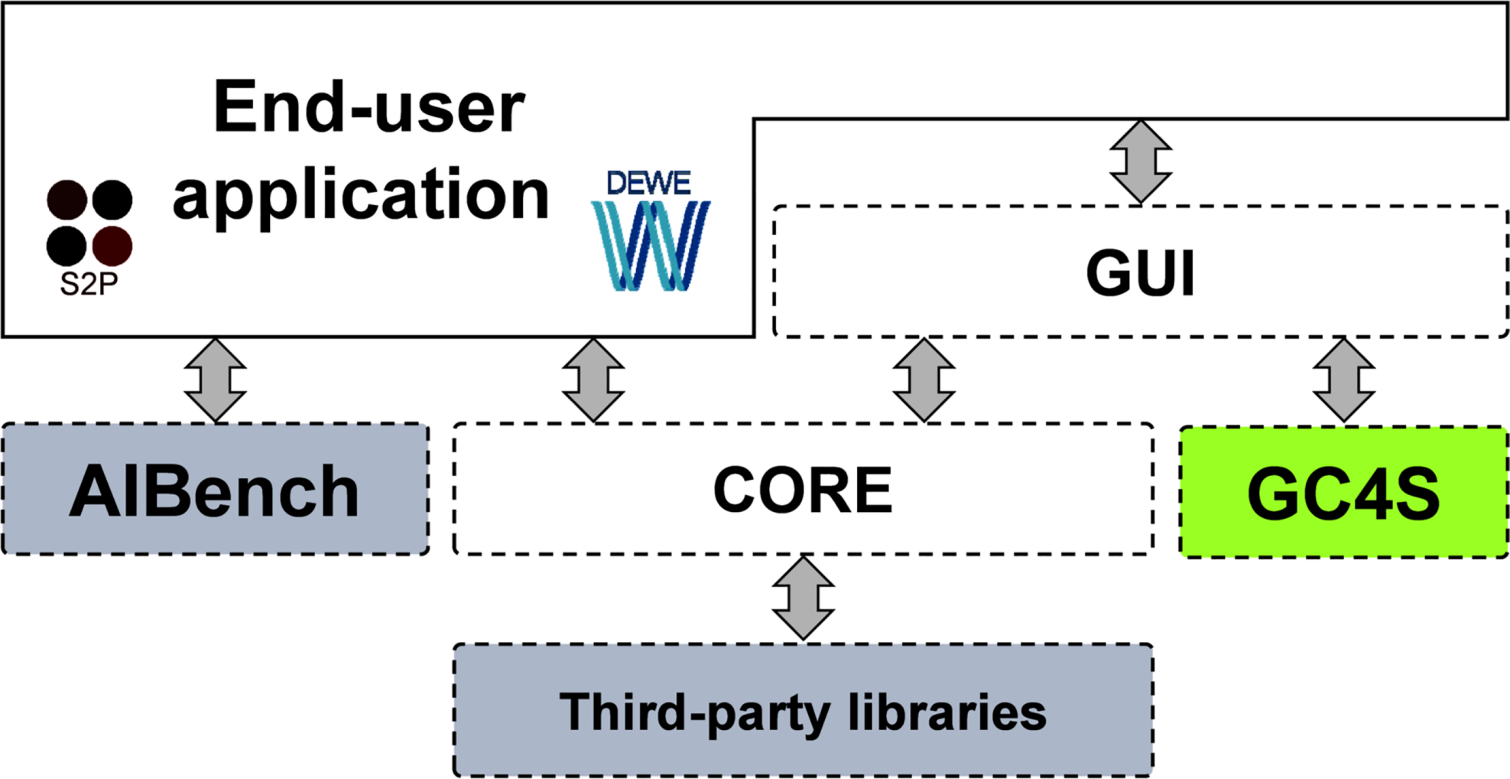
AIBench-based applications architecture (S2P and DEWE).

S2P (http://www.sing-group.org/s2p) and DEWE (http://www.sing-group.org/dewe) were constructed using the AIBench framework following the straightforward architecture depicted in Fig 10. The *gui* module of both applications was entirely constructed with GC4S, allowing us to focus on the specific needs of those developments relying on the general purpose components offered by GC4S.

**Fig 11:**
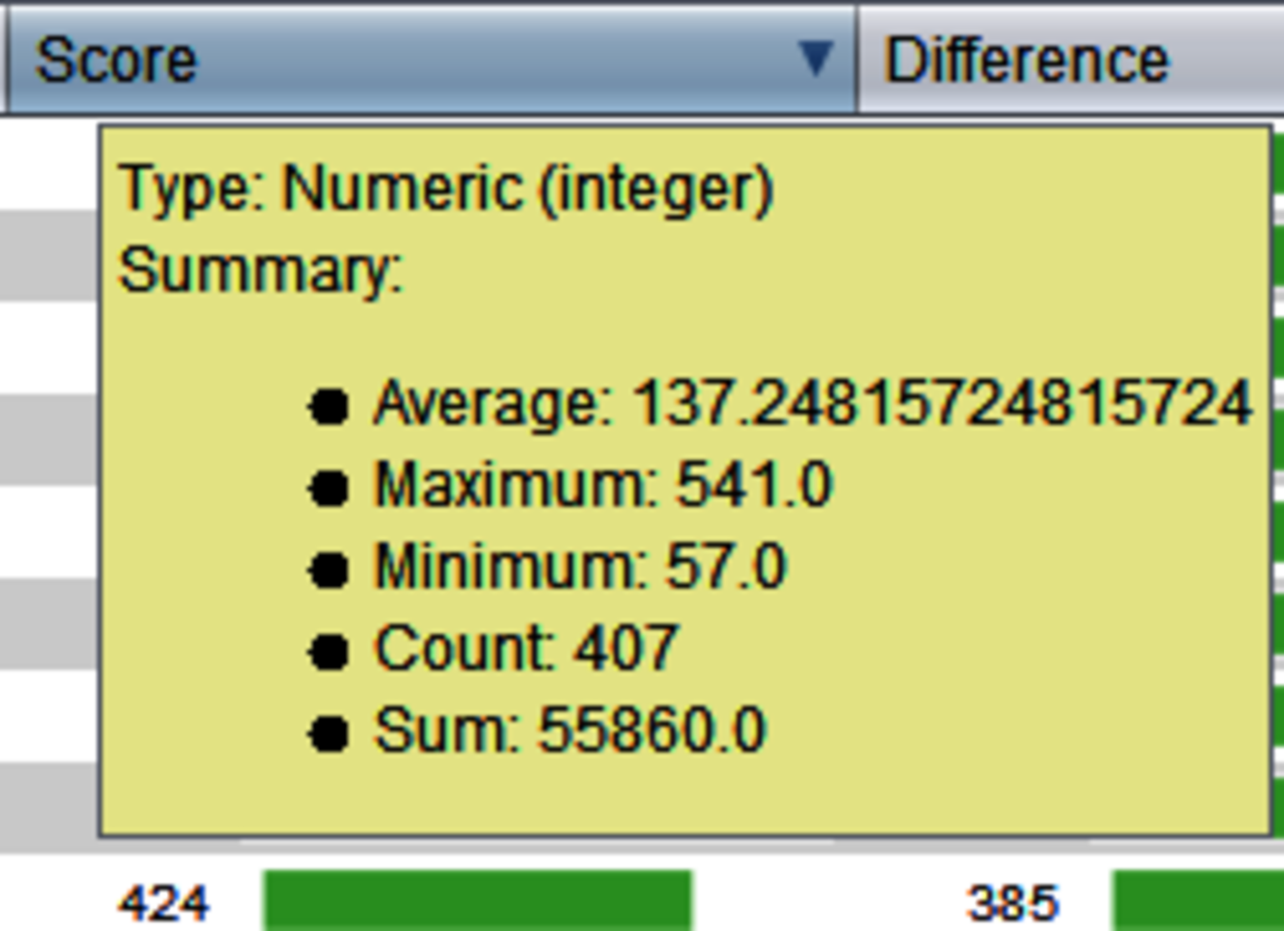
A popup summary added to each column of a table using the ColumnSummaryTableCellRenderer component.

**Fig 12:**
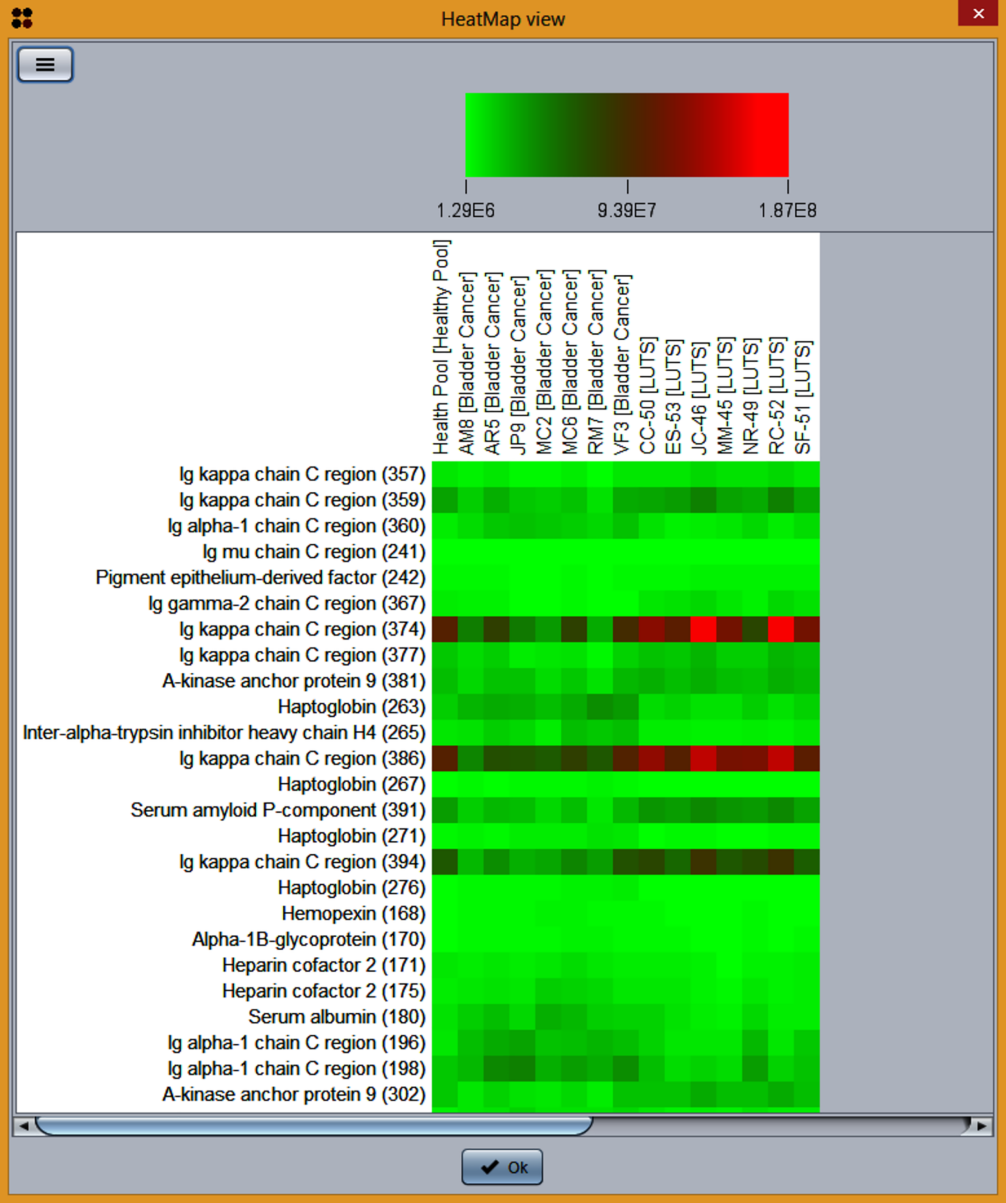
Heat map visualization of spots data in S2P using the JHeatMap component.

**Fig 13:**
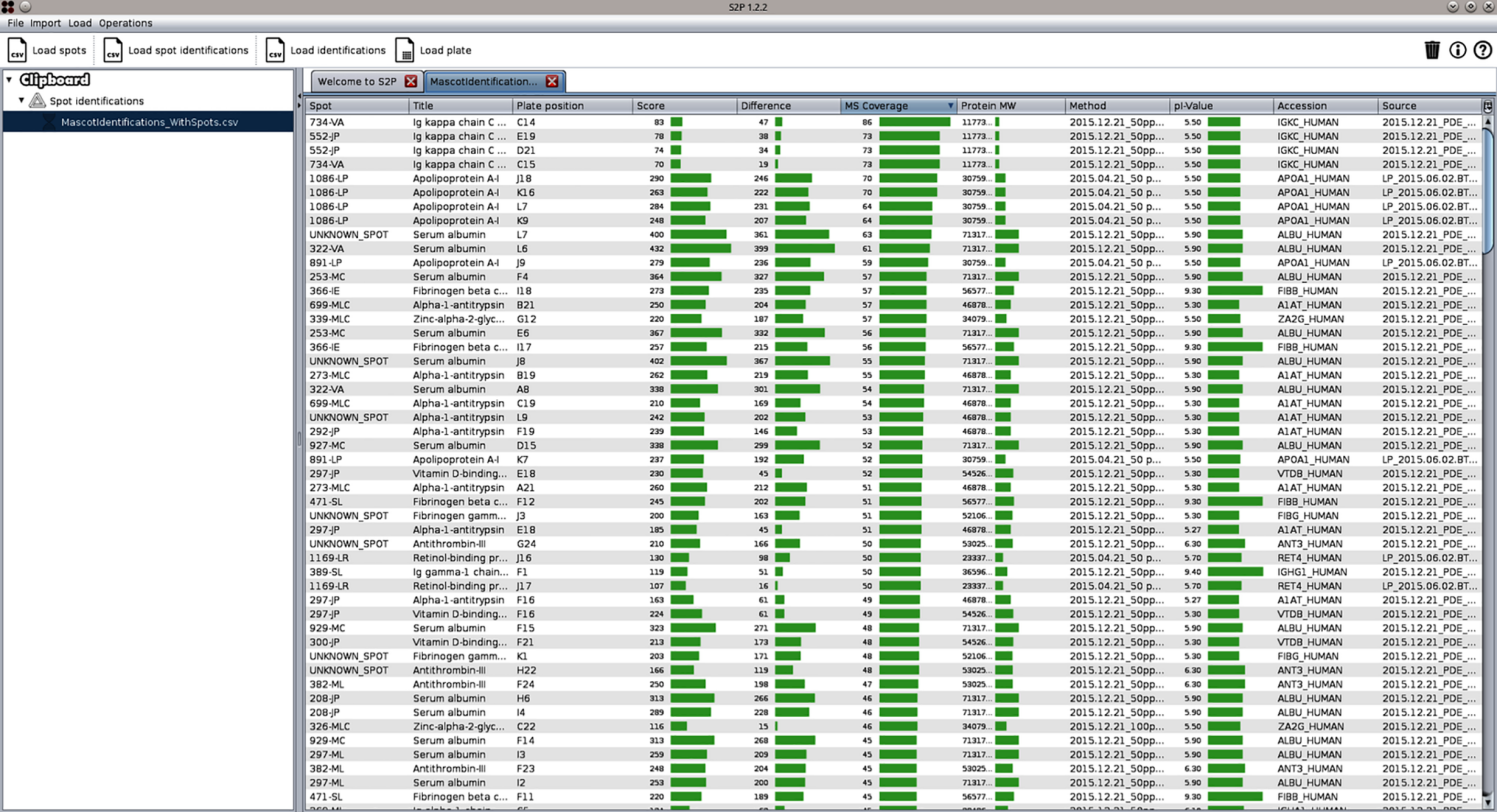
Screenshot of the S2P application showing the table that displays a Mascot identifications report. This and other tables are enhanced with spark lines from the JSparklines library that are created using the *gc4s-jsparklines-factory* module.

S2P is an open source application for fast and accurate processing of 2D-gel and MALDI-based mass spectrometry protein data. Since this application allows users to manage and visualize different types of available data, GC4S was used to provide a rich and effective user experience. For instance, GC4S allowed enhancing tables by adding a popup summary to each column (Fig 11) as well as the creation of interactive and customizable heat map visualizations (Fig 12). Tables are also enhanced by adding spark lines from the JSparklines library that are created using the *gc4s-jsparklines-factory* module (Fig 13).

**Fig 14:**
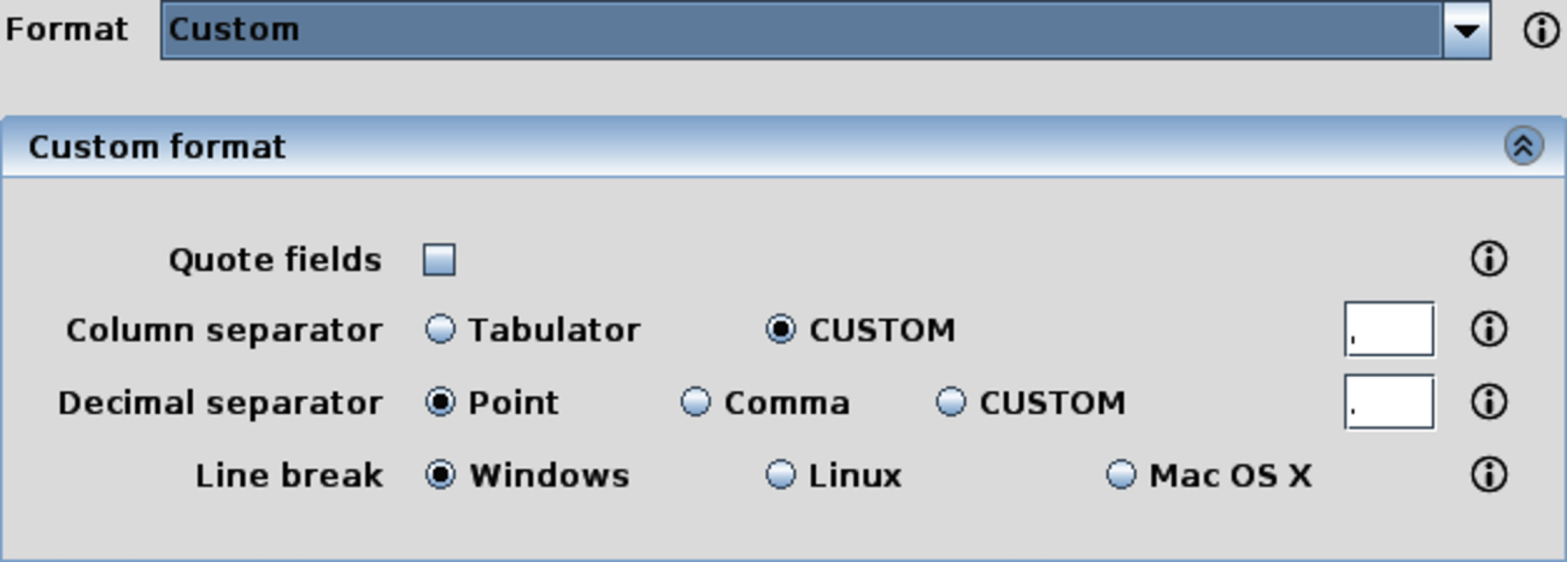
The CsvPanel component allows users to configure their own CSV format.

Additionally, S2P also makes intensive use of the previously mentioned InputParametersPanel component to retrieve user inputs for different operations. Regarding this functionality, there is a noteworthy case: S2P deals with comma-separated values (CSV) files, and it must retrieve the CSV format configuration from the user. As this format is application-independent, GC4S offers a component called CsvPanel, which allows users to either customize or choose a predefined CSV format (Fig 14).

DEWE is a novel open source application for executing RNA-Seq analysis focused on supporting differential expression experiments. As in the case of S2P, this application also benefits from GC4S components to provide a compelling user experience, using the table enhancements previously discussed together with the InputParametersPanel.

**Fig 15:**
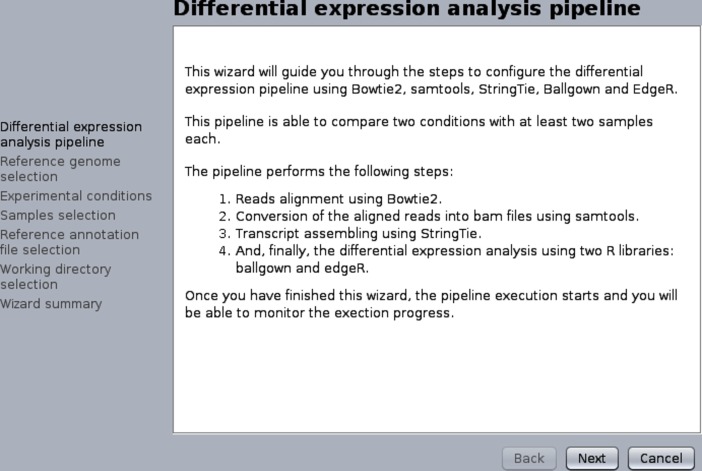
Example of use of a Wizard in DEWE to create a workflow configuration assistant.

The DEWE project has the specific goal of facilitating the configuration and execution of differential expression analysis workflows. As these workflows require different inputs and configurations, a configuration assistant or wizard seemed a good way of supporting this functionality. Consequently, the Wizard component was included in the GC4S library to facilitate the creation of such configuration assistants. This particular component extends JDialog and accepts a list of WizardStep objects. Fig 15 shows the configuration assistant of one of the built-in workflows included in DEWE. While DEWE only needs to implement the specific WizardStep components needed to configure the workflow (i.e. the configuration steps), the Wizard component implemented in GC4S constructs the assistant dialog and manages the wizard buttons as well as the left sidebar that contains the steps labels.

### Standalone developments

**Fig 16:**
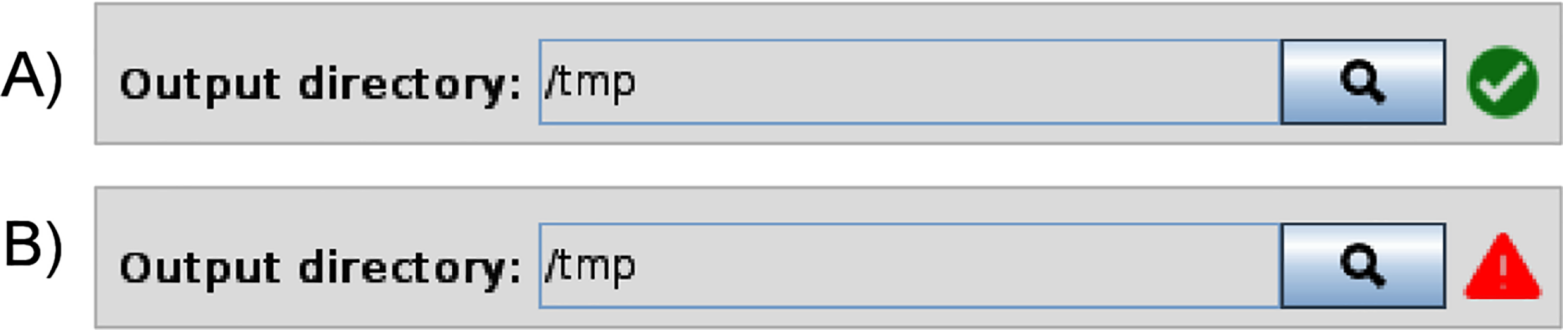
Example of use of different icons provided by the Icons class.

As previously commented, the components of the GC4S library can be used either separately or in conjunction with the AIBench framework to implement the view components. An illustrative example of the former case is the development of SEDA (SEquence DAtaset Builder). SEDA (http://www.sing-group.org/seda) is Java Swing application for efficient and flexible processing of FASTA files, offering different functionalities. Apart from using some of the previously commented components, two other notable examples illustrate the broad scope of our GC4S library. The first example involves the use of some of the icons provided in the Icons class. For instance, Fig 16 shows how a different icon from GC4S is used depending on the validity of the selected output directory, which is done using a JFileChooserPanel component.

**Fig 17:**
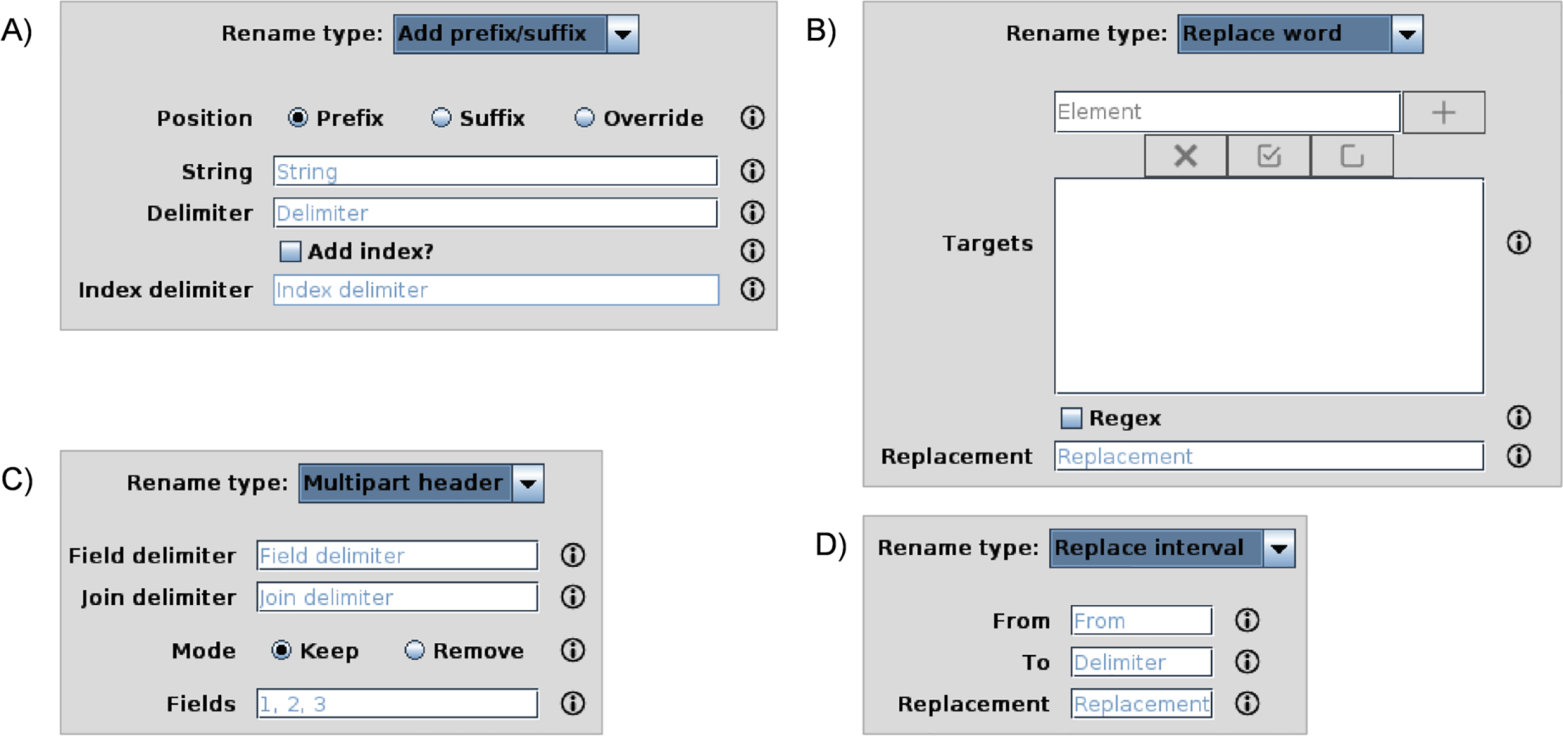
**Use of CardsPanel in SEDA to display a different configuration panel (A, B, C or D) to the user depending on the choice made in the *Rename type* combo box**.

The second example involves a very common situation in which a different panel needs to be shown to the user depending on a previous selection in a combo box. This can be easily achieved by using a CardLayout, switching the visible panel (or *card*) when the user selection changes. The CardsPanel component provides this functionality and it is used in SEDA to display different configuration panels to the user, depending on the selection previously made in a combo box (Fig 17).

### Discussion and lessons learned

Two of the main forms of providing reusable software include libraries and frameworks. While libraries can be seen as a set of reusable data structures and functions, frameworks incorporate the concept of *dependency inversion* whereby the framework shapes the architecture of the application and the user code is *plugged* into the framework to build the final application. Libraries and frameworks are two complementary concepts. While frameworks usually incorporate libraries with helper functions, they are different concepts.

Java is a good ecosystem for developing reusable software. Not only because of its object oriented nature, which eases reusability by taking advantage of encapsulation and polymorphism, but also because of (*i*) Swing, which includes a JComponent base class for every visual component and a delegation event model that allows programmers to create and reuse custom interactive components easily, and (*ii*) Maven, which speeds-up the incorporation of libraries and frameworks to new projects due to its dependency management system.

The Java ecosystem incorporated in 2008 the JavaFX toolkit for the creation of GUI desktop applications as well as Rich Internet Applications. It was meant to be the successor of Java Swing because of its modern features (such as built-in UI controls, CSS support, the WebView component, or smooth animations, among others), but it seems that it has not met the expectations, as it has not achieved the same adoption and support levels (in terms of community forums and experts, for instance) than Java Swing. Furthermore, the sophisticated GUI features included in JavaFX may not be so essential for the development of scientific applications that are easy to use for end-users without advanced informatics skills, while the relative simplicity of Swing probably makes it more appropriate for academia. Although it is hard to estimate the actual usage of both libraries, some numbers can be obtained in this regard. By July 2018 we have seen that: (*i*) a Google Scholar query shows 42 400 entries for “Java Swing” and 4 950 for “JavaFX”, (*ii*) a generic Google search shows about 26 600 000 results for “Java Swing” and about 4 170 000 for “JavaFX”, and (*iii*) a Scopus query throws only 40 results for “JavaFX” but 258 for “Java Swing”. Based on this numbers and the maturity of the Java Swing technology, for which there are many additional third-party libraries and frameworks, it is still an appropriate choice for the development of scientific and bioinformatics software.

In this paper we have presented GC4S, a library of reusable Java Swing components, which is a very useful complement to our AIBench framework for biomedical desktop applications. While AIBench provides applications with a reusable main architecture and structure, based on the input-process-output (IPO) model, the GC4S library provides a set of components that are incorporated as needed for each specific application.

**Fig 18:**
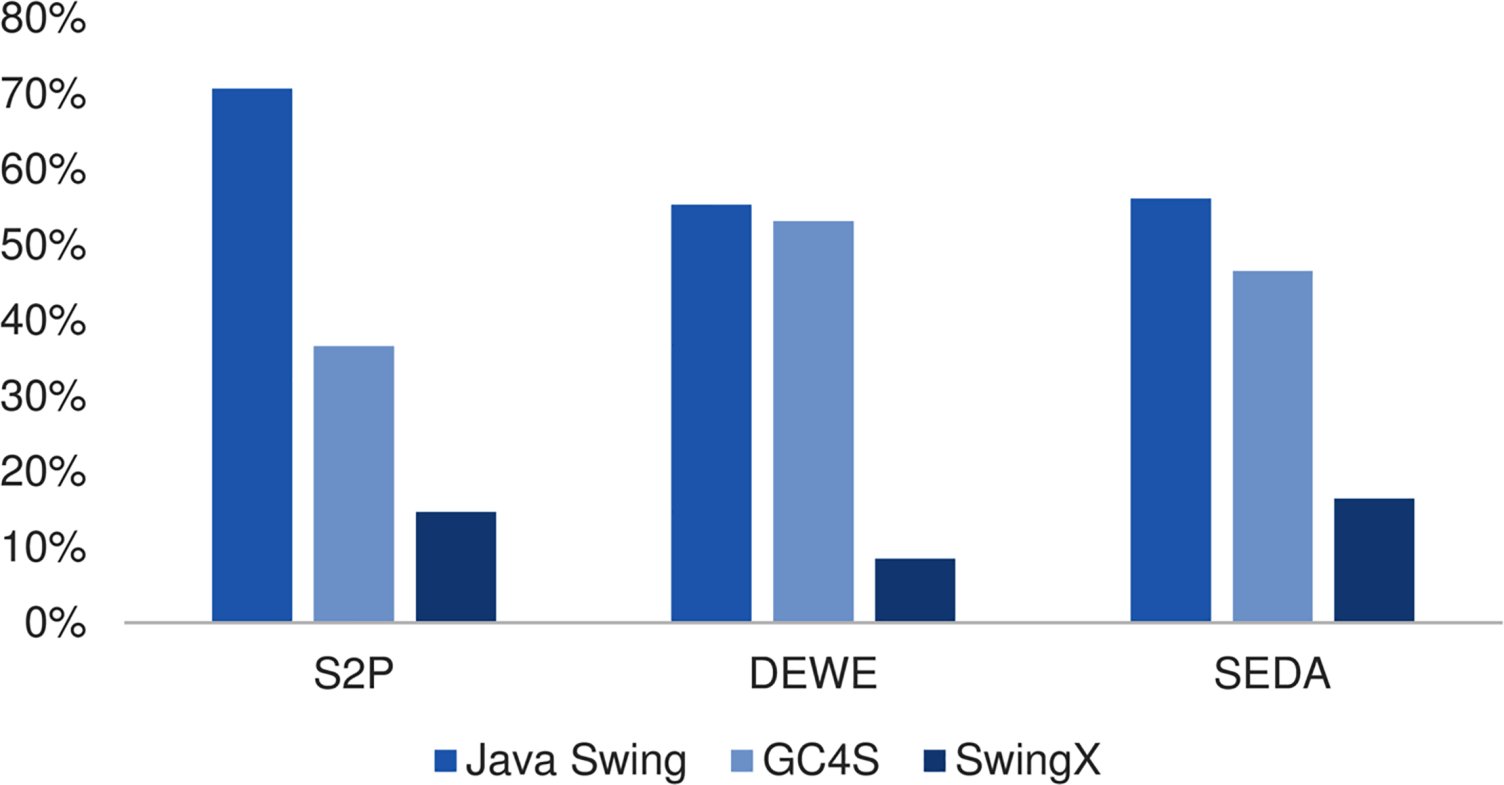
Percentages of GUI classes in S2P, DEWE and SEDA with references to Java Swing, GC4S and SwingX classes.

We provided an overview of the GC4S library and presented three real-world use cases to illustrate its capabilities and usefulness. When developing the GUI of these three software applications, Java Swing, SwingX and GC4S were used. As seen in Fig 18, Java Swing classes are the most referenced by GUI classes in the three projects. Nevertheless, the percentage of references to GC4S is notable, especially in DEWE and SEDA. This demonstrates the impact that the presented library had on the development of these three applications.

## Conclusions

GC4S (http://www.sing-group.org/GC4S) is open-source and freely distributed under license LGPLv3, providing a set of reusable graphical user interface components for Java Swing. The real utility of GC4S has been demonstrated by different case studies where its use has led to a more efficient software development process. GC4S is open to further extensions and we will keep improving it with fresh and innovative ideas as existing projects evolve or novel developments reach the marketplace.

## Supporting information

S1 Table Complete list of GC4S components by package and type. Packages and components are listed alphabetically.
(DOCX)

S1 Document Basic examples of GC4S. (DOCX)
