# Supplementary material for: GC4S: A bioinformatics-oriented Java software library of reusable graphical user interface components
Source: PLoS One. 2018 Sep 20;13(9):e0204474. doi: 10.1371/journal.pone.0204474 (PMC6147514; doi:10.1371/journal.pone.0204474)
Supplement: S1 Table — Packages and components are listed alphabetically. (DOCX) [file pone.0204474.s001.docx]

| **Package** | **Component** | **Type** | **Description** |
| --- | --- | --- | --- |
| *.dialog | AbstractInputJDialog | Input | An abstract class for creating input dialogs. It is widely used in GC4S as well as in external projects. |
|  | ColorsSelectionDialog | Input | A dialog that allows a user to select a list of colors using a ColorsListPanel. |
|  | ExportCsvDialog | Input | An input dialog that allows the configuration of a CSV format. Internally, it uses a CsvPanel. |
|  | FontConfigurationDialog | Input | An input dialog that uses a FontConfigurationPanel component to allow users selecting a Font. |
|  | JOptionPaneMessage | Utility | Represents a message for a JOptionPane and adds a check box to allow the user to not show the message again. |
|  | JProgressDialog | Visualization | Facilitates the creation of progress dialogs based on fixed task lists. |
|  | ListSelectionDialog<E> | Input | An input dialog that uses a JParallelListsPanel component to retrieve user selection. |
|  | WorkingDialog | Visualization | Shows a progress in work dialog with an indeterminate progress bar and a label. The indeterminate progress bar may be replaced by a user image. |
| *.dialog.wizard | Wizard | Input | A wizard dialog that allows showing a list of WizardStep. |
|  | WizardStep | Input | An abstract class that represents a step in a Wizard. Concrete steps must implement abstract methods in this class in order to allow the Wizard dialog constructing the assistant |
| *.dialog.wizard.event | WizardStepAdapter | Utility | An abstract adapter class for receiving wizard step events. The methods in this class are empty. This class exists as convenience for creating WizardStepListener objects. |
|  | WizardStepEvent | Utility | A WizardStepEvent is used to notify interested parties that state has changed in the WizardStep source. |
|  | WizardStepListener | Utility | The listener interface for receiving wizard step events. |
| *.event | AbstractKeyPressedListener | Utility | An abstract implementation of KeyListener that runs an action when a specific key is pressed. |
|  | DocumentAdapter | Utility | An abstract adapter class for receiving text document events. The methods in this class are empty. This class exists as convenience for creating listener objects. |
|  | EnterKeyPressedListener | Utility | A concrete implementation of AbstractKeyPressedListener} to listen for enter key. |
|  | ListDataAdapter | Utility | An abstract adapter class for receiving data list events. The methods in this class are empty. This class exists as convenience for creating listener objects. |
|  | PopupMenuAdapter | Utility | An abstract adapter class for receiving popup menu events. The methods in this class are empty. This class exists as convenience for creating listener objects. |
|  | TextFieldSelectionFocusListener | Utility | A FocusAdapter implementation that selects all text in a JTextField when it gains focus |
| *.input | DoubleRange | Utility | This class represents a range (i.e. a pair of a minimun and a maximum) of Double. |
|  | DoubleRangeInputDialog | Input | A dialog that allows user to introduce a range of double values using a DoubleRangeInputPanel. |
|  | DoubleRangeInputPanel | Input | A panel that allows user to type a range of double values. |
|  | DoubleRangeSpinnerInputPanel |  | A panel that allows user to type a range of double values using spinners. |
|  | FontConfigurationPanel | Input | A panel that allows user to configure a font. |
|  | InputParameter | Utility | This class encapsulates three components of an input parameter: a description label, a JComponent to use to retrieve user input, and a label to use as help for this parameter. |
|  | InputParameterPanel | Input | A panel that displays one or more InputParameter objects. |
|  | ItemSelectionDialog<T> | Input | A dialog that allows users choosing *n* items from different combo boxes using an ItemSelectionPanel. |
|  | ItemSelectionPanel<T> | Input | A panel that allows users choosing *n* items from different combo boxes. |
|  | JColorChooserButton | Input | A button that allows users selecting a color by using a JColorChooser. |
|  | RadioButtonsPanel<T> | Input | A component that allows users selecting a single item from a list of items using radio buttons. |
|  | RangeInputPanel | Input | A panel that allows user selecting a range of values, taking into account that the minimum value should always be lower or equal to the maximum value. It uses JSlider components for receiving user inputs. |
| *.input.combobox | ExtendedJCombobox<T> | Input | An extension of JComboBox that allows specifying whether the size of the combo box must be adapted to its items width when the drop down list is being displayed |
|  | ComboBoxItem<T> | Utility | Wraps any object so that it can be easily added to a JComboBox. |
| *.input.csv | CsvFormat | Utility | Specifies the format of a CSV file. |
|  | CsvPanel | Input | A panel that allows user to configure a CSV format. |
| *.input.csv.event | CsvListener | Utility | The listener interface for receiving CSV events. |
| *.input.filechooser | ExtensionFileFilter | Utility | A concrete implementation FileFilter that provides the possibility of filtering files based on a regular expression. It also allows choosing whether the regular expression must be evaluated as case-sensitive or not. |
|  | JFileChooserConfiguration | Utility | A class that encapsulates the configuration of a JFileChooser. |
|  | JFileChooserPanel | Input | Displays a text field together a browse button that allows users to select a file. The name of the file selected is shown in the text field. |
|  | JFileChooserPanelBuilder | Utility | A builder class for JFileChooserPanel. |
|  | JMultipleFileChooserPanel | Input | Displays a JList along with a browse button that allows users to select several files. The names of the selected files are shown in the list. |
|  | JMultipleFileChooserPanelBuilder | Utility | A builder class for JMultipleFileChooserPanel. |
|  | Mode | Utility | The mode to open a JFileChooser. |
|  | SelectionMode | Utility | The selection mode of a JFileChooser. |
| *.input.filechooser.event | FileChooserListener | Utility | The listener interface for receiving JFileChooserPanel events. |
|  | MultipleFileChooserListener | Utility | The listener interface for receiving JMultipleFileChooserPanel events. |
| *.input.list | ColorsListPanel | Input | This class encloses a JListPanel<Color> to provide the ability of adding Color elements to the list. |
|  | ExtendedDefaultListModel<E> | Utility | This class extends DefaultListModel to add move down/up element and add several elements functionalities. |
|  | FilteredListModel<E> | Utility | This class extends AbstractListModel and allows filtering a given ListModel. |
|  | JInputList | Input | A class that encloses a JListPanel of String in order to provide the ability of adding elements to the list. |
|  | JListPanel<E> | Input | This class encloses a JList based on a ExtendedDefaultListModel to provide common list functionalities (moving elements, select all, clear selection, filtering elements). |
|  | JParallelListsPanel<E> | Input | A JParallelListsPanel displays two parallel JListPanel allowing to move elements between them. |
| *.input.list.event | DefaultListDataListener | Utility | A default implementation of ListDataListener. |
|  | ExtendedDefaultListModelListener | Utility | The listener interface for receiving ExtendedDefaultListModel events. |
| *.input.text | BindJXTextField | Input | An extension of JXTextField that allows to specify a function to consume text field's value when it changes. |
|  | DoubleTextField | Input | An extension of JFormattedTextField to create text fields for allow the input of double values. |
|  | ExtendedJXTextField | Input | An extension of JXTextField that provides additional features. |
|  | IntegerRangeInputPanel | Input | A panel that allows user typing a range defined by values, taking into account that the minimum value should always be lower or equal to the maximum value. |
|  | JIntegerTextField | Input | An extension of JFormattedTextField that only accepts integers as input. |
|  | JLimitedTextField | Input | An extension of JTextField that allows specifying the maximum extension of the document. |
| *.ui | CardsPanel | UI | A JPanel that displays different components using a CardLayout and creates a combo box to control which one should be visible. |
|  | CardsPanelBuilder | Utility | A builder class for CardsPanel. |
|  | CenteredJPanel | UI | A JPanel that only accepts one Component and displays it in the center. |
|  | ColorListCellRenderer | UI | An implementation of ListCellRenderer to show colors. |
|  | ComponentsListPanel | UI | A component that allows showing a list of generic components with control buttons to add and remove them. |
|  | Orientation | Utility | An enumeration that defines possible orientation of components. |
|  | UIUtils | Utility | Provides functionalities to deal with UI components |
| *.ui.icons | ColorIcon | UI | Draws a square of the established size and color. |
|  | Icons | UI | Provides a set of icons in different sizes. |
|  | IconsPanel | Utility | Shows all icons in the Icons class. |
| *.ui.menu | ExtendedJSeparator | UI | An extension of JSeparator that allows specifying a margin for the component. |
|  | HamburgerMenu | UI | A hamburger button to display a menu. |
| *.ui.tabbedpane | ButtonTabComponent | UI | A component to use as tab component. It contains a JLabel to show the text and a JButton to close the tab it belongs to. |
|  | CloseableJTabbedPane | UI | An extension of JTabbedPane to add close buttons to tabs. |
|  | ExtendedJTabbedPane | UI | An extension of JTabbedPane that adds useful features such as the possibility of tell the tabbed pane to hide the tab bar when there is only one tab. |
| *.ui.text | MultilineLabel | UI | An extension JTextArea configured as multiline label. |
|  | VerticalLabelUI | UI | An UI to display vertical text in JLabel components. |
| *.utilities | Checks | Utility | A class that provides additional checks to use in component constructors. |
|  | ExtendedAbstractAction | Utility | An extension of AbstractAction to reduce verbosity when creating actions by allowing to construct actions by passing references to methods without parameters. |
|  | FileDrop | Utility | A class to facilitate drag and drop files from the operating system. |
|  | FileDropListener | Utility | Interface to listen when files are dropped. |
|  | FontUtils | Utility | Provides functionalities to deal with fonts. |
|  | Gradient | Utility | Provides functions to create color gradients. |
|  | ImageIOUtils | Utility | Provides functionalities to deal with ImageIO. |
|  | MatrixUtils | Utility | Provides functionalities to deal with data matrices. |
|  | StringMetrics | Utility | A utility class to compute the size of a String given a Graphics2D and a Font. |
| *.utilities.builder | JButtonBuilder | Utility | A builder class to construct buttons. |
|  | JToggleButtonBuilder<T extends JToggleButton> | Utility | A builder class to construct toggle buttons. |
| *.visualization | ColorKeyLegend | Visualization | A component that draws a color key map between two colors. |
|  | ColorLegend | Visualization | A component that shows a legend of colors and their associated labels. |
|  | VisualizationUtils | Utility | Utility methods for showing components. |
| *.visualization.heatmap | JHeatMap | Visualization | Provides a heat map representation for a double matrix. |
|  | JHeatMapDataOperationsDialog | Input | An input dialog that shows the different operations that can be applied to a heat map. |
|  | JHeatMapModel | Utility | This class encapsulates the data needed by JHeatMap. |
|  | JHeatMapOperations | Utility | Stores different types operations that can be applied to heat map data. |
|  | JHeatMapPanel | Visualization | Wraps a JHeatMap adding a toolbar with options to manipulate it. |
| *.visualization.table | BeanTableModel<T> | Utility | A table model for easily displaying a list of Java beans in a table, using one row for each bean and one column for each bean's property. |
|  | ColumnSummaryTabeCellRenderer | Visualization | A table cell renderer that displays a summary of all values of the column that the cell to be rendered belongs. This renderer is intended to be used in JTableHeader and can render summaries for columns containing objects of the following classes: String, Double, Float, Integer, Long and File. |
|  | ExtendedDefaultTableModel | Utility | An extension of the DefaultTableModel that provides an implementation of the DefaultTableModel#getColumnClass(int). |
|  | ExtendedJXTable | Visualization | An extension of JXTable that allows to hide/show the column visibility actions and facilitates adding own actions to the ColumnControlButton. |
|  | FilterableJXTable | Visualization | An extension of JXTable that allows establishing a row filter. |
|  | RowHeaderExtendedJXTable | Visualization | A RowHeaderExtendedJXTable extends a ExtendedJXTable to allow users creating tables within a row names column. |
|  | RowHeaderTableModel | Utility | A RowHeaderTableModel wraps a TableModel adding row names. |
|  | TableUtils | Utility | Provides functionalities to deal with tables. |
