# Supplementary material for: GC4S: A bioinformatics-oriented Java software library of reusable graphical user interface components
Source: PLoS One. 2018 Sep 20;13(9):e0204474. doi: 10.1371/journal.pone.0204474 (PMC6147514; doi:10.1371/journal.pone.0204474)
Supplement: S1 Document — (DOCX) [file pone.0204474.s002.docx]

# Using GC4S in Maven-based projects

To use GC4S in a Maven-based Java project, developers need only declare dependency in the pom.xml file of the project (Fig S1.1), along with our official maven repository, where it is available. The artifactId must be the identifier of one of the six GC4S modules. Since modules gc4s-genomebrowser, gc4s-heatmap, gc4s-jsparklines-factory, gc4s-multiple-sequence-alignment-viewer and gc4s-statistics-tests-table depend on the general gc4s module, they also provide access to it without needing to declare it explicitly.


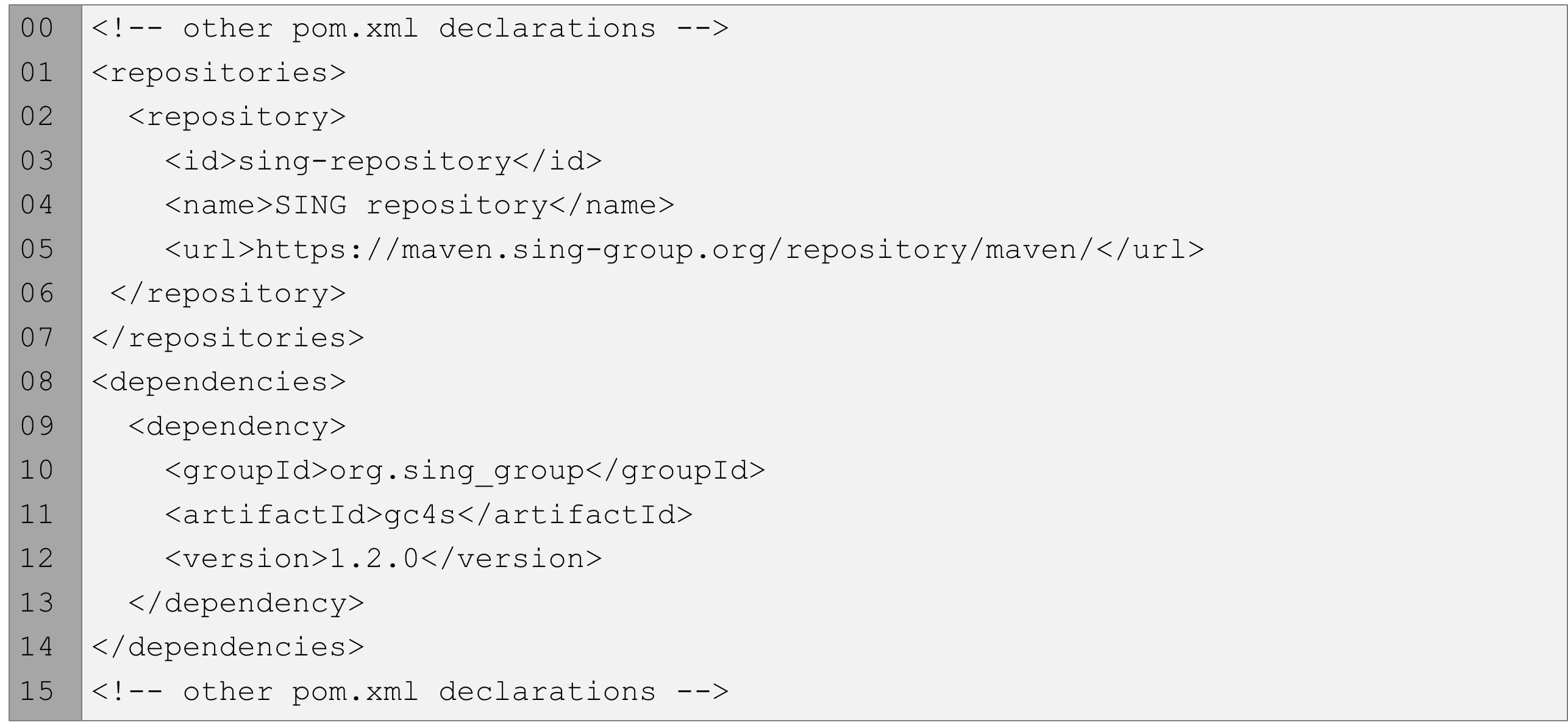


**Fig S1.1. GC4S maven dependency.**

# *gc4s* module examples

These section presents two simple but illustrative examples that are in line with the overall purpose of all the components included in the gc4s module of our library. A comprehensive set of usage examples such as that presented above can be found in the *gc4s-demo* module, covering each component of the module.

## JFileChooserPanel

The appropriate way of selecting a file in Java Swing is to use a JFileChooser. When retrieving user inputs, in addition to selecting a given file, it is also frequent to show the selected file along with a browse button to use the JFileChooser. In order to do so, we developed a specific JFileChooserPanel, which was in fact the first GC4S component created.


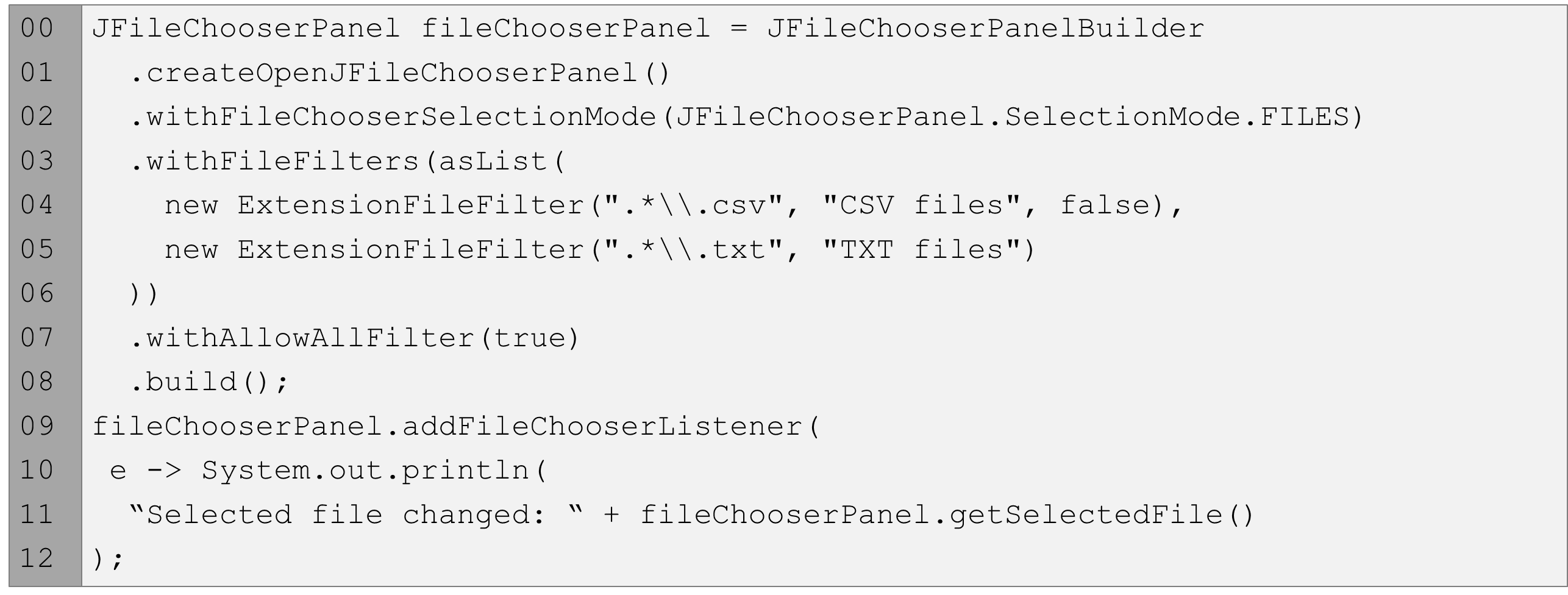


**Fig S1.2. Code snippet showing the instantiation of an JFileChooserPanel. Also, a listener is added in order to know when the file selection changes and to show the selected file.**

The code snippet shown in Fig S1.2 illustrates the creation of the JFileChooserPanel presented in Fig S1.3A using a JFileChooserBuilder, which facilitates the creation of complex configurations. In this illustrative example, the component is configured with three file filters.


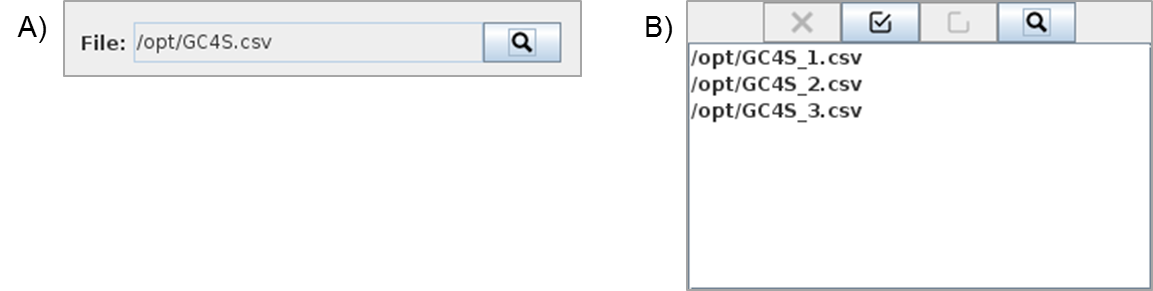


**Fig S1.3. GC4S components for file selection: A) the JFileChooserPanel component, B) the JMultipleFileChooserPanel component.**

Similarly, the JMultipleFileChooserPanel component shown in Fig S3.3B can be used when the selection of several files is needed.

## InputParametersPanel

The creation of configuration panels to obtain a set of parameter-value pairs is also a common scenario that developers face continuously. In such panels, there are three entries of each input parameter that must be shown to the user: (*i*) the description label; (*ii*) the actual component that retrieves the user input (e.g. a text field); and (*iii*) a description or help message that gives more information about the parameter. In GC4S, this information is encapsulated into an object of class InputParameter. Accordingly, the panel that shows one or more objects of this class is an InputParametersPanel.

The code snippet shown in Fig S1.4 illustrates the creation of an InputParametersPanel with two sample parameters shown in S1.5. The use of this particular component provides two advantages: (*i*) a consistent interface across different configuration components of the application; and (*ii*) when using AIBench, a similar interface compared to the one in the automatically generated dialogs.


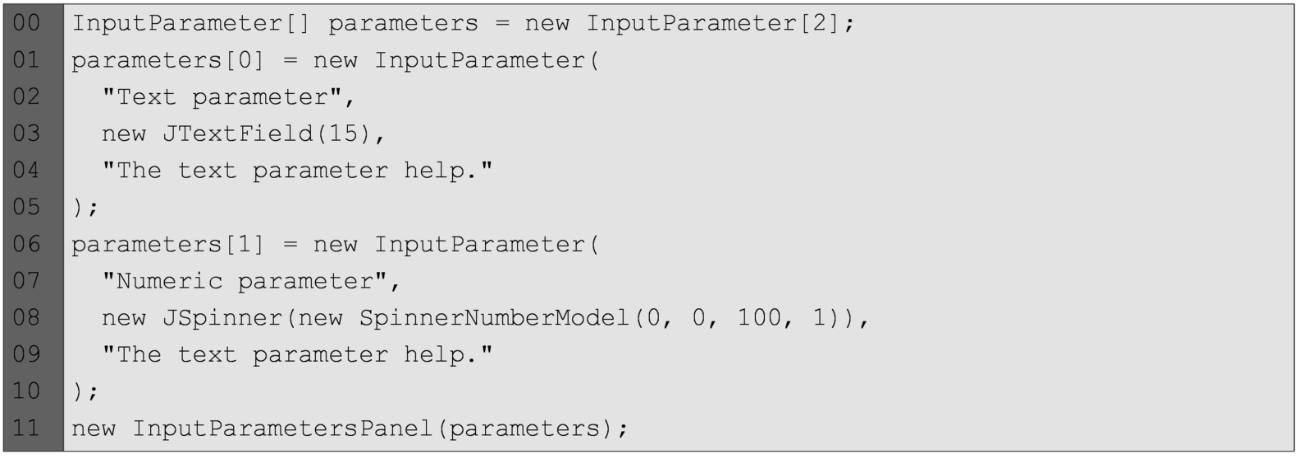


**Fig S1.4. Code snippet showing the instantiation of an InputParametersPanel.**


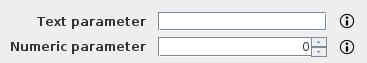


**Fig S1.5. The InputParametersPanel component created by the code snippet shown in Fig S1.4.**
